# Efficacy and Safety of Angiotensin-Converting Enzyme Inhibitor in Combination with Angiotensin-Receptor Blocker in Chronic Kidney Disease Based on Dose: A Systematic Review and Meta-Analysis

**DOI:** 10.3389/fphar.2021.638611

**Published:** 2021-05-06

**Authors:** Mingming Zhao, Rumeng Wang, Yi Yu, Meiying Chang, Sijia Ma, Hanwen Zhang, Hua Qu, Yu Zhang

**Affiliations:** ^1^ Department of Nephrology, Xiyuan Hospital, China Academy of Chinese Medical Sciences, Beijing, China; ^2^ Beijing University of Chinese Medicine, Beijing, China; ^3^ Department of Statistics, Purdue University, West Lafayette, IN, America; ^4^ Xiyuan Hospital, China Academy of Chinese Medical Sciences, Beijing, China; ^5^ NMPA Key Laboratory for Clinical Research and Evaluation of Traditional Chinese Medicine, Beijing, China; ^6^ National Clinical Research Center for Chinese Medicine Cardiology, Beijing, China

**Keywords:** ACEI in combination with ARB, dose, chronic kidney disease, urine albumin excretion, urine protein excretion, glomerular filtration rate, hyperkalemia, hypotension

## Abstract

**Background:** The purpose of this meta-analysis was to evaluate the controversy of angiotensin-converting enzyme inhibitor (ACEI) in combination with angiotensin-receptor blocker (ARB) in the treatment of chronic kidney disease (CKD) based on dose.

**Methods:** PubMed, EMBASE, and Cochrane Library were searched to identify randomized controlled trials (RCTs) from inception to March 2020. The random effects model was used to calculate the effect sizes. Potential sources of heterogeneity were detected using sensitivity analysis and meta-regression.

**Results:** This meta-analysis of 53 RCTs with 6,375 patients demonstrated that in patients with CKD, ACEI in combination with ARB was superior to low-dose ACEI or ARB in reducing urine albumin excretion (SMD, −0.43; 95% CI, −0.67 to −0.19; *p* = 0.001), urine protein excretion (SMD, −0.22; 95% CI, −0.33 to −0.11; *p* < 0.001), and blood pressure (BP), including systolic BP (WMD, −2.89; 95% CI, −3.88 to −1.89; *p* < 0.001) and diastolic BP (WMD, −3.02; 95% CI, −4.46 to −1.58; *p* < 0.001). However, it was associated with decreased glomerular filtration rate (GFR) (SMD, −0.13; 95% CI, −0.24 to −0.02; *p* = 0.02) and increased rates of hyperkalemia (RR, 2.07; 95% CI, 1.55 to 2.76; *p* < 0.001) and hypotension (RR, 2.19; 95% CI, 1.35 to 3.54; *p* = 0.001). ACEI in combination with ARB was more effective than high-dose ACEI or ARB in reducing urine albumin excretion (SMD, −0.84; 95% CI, −1.26 to −0.43; *p* < 0.001) and urine protein excretion (SMD, −0.24; 95% CI, −0.39 to −0.09; *p* = 0.002), without decrease in GFR (SMD, 0.02; 95% CI, −0.12 to 0.15; *p* = 0.78) and increase in rate of hyperkalemia (RR, 0.94; 95% CI, 0.65 to 1.37; *p* = 0.76). Nonetheless, the combination did not decrease the BP and increased the rate of hypotension (RR, 3.95; 95% CI, 1.13 to 13.84; *p* = 0.03) compared with high-dose ACEI or ARB.

**Conclusion:** ACEI in combination with ARB is superior in reducing urine albumin excretion and urine protein excretion. The combination is more effective than high-dose ACEI or ARB without decreasing GFR and increasing the incidence of hyperkalemia. Despite the risk of hypotension, ACEI in combination with ARB is a better choice for CKD patients who need to increase the dose of ACEI or ARB **(**PROSPERO CRD42020179398).

## Introduction

Chronic kidney disease, characterized by a reduced glomerular filtration rate (GFR) and/or increased urinary albumin excretion, is an increasing public health issue owing to its high prevalence and increased risk of end-stage renal disease, cardiovascular disease, and premature death (Matsushita et al., 2010). The prevalence of CKD is estimated to be 8–16% worldwide (Jha et al., 2013). CKD is a great global-health challenge, especially in low- and middle-income countries (Mills et al., 2015). National and international efforts for the prevention, detection, and treatment of CKD are needed to reduce its morbidity and mortality worldwide.

Hypertension commonly coexists with CKD, and its prevalence progressively increases with decline in kidney function (Muntner et al., 2010; Egan et al., 2014). According to recent guidelines, angiotensin-converting enzyme inhibitor (ACEI) or angiotensin-receptor blocker (ARB) should be the drugs of first choice for CKD (Kalaitzidis and Elisaf, 2018). The 2020 Kidney disease: Improving Global Outcomes (KDIGO) guideline recommends that treatment with an ACEI or an ARB be initiated in patients with diabetes, hypertension, and albuminuria and that these medications be titrated to the highest approved dose that is tolerated. The 2012 KDIGO guideline on IgA nephropathy recommends long-term ACEI or ARB treatment when proteinuria is >1 g/d, with up-titration of the drug depending on blood pressure (BP), and to achieve proteinuria <1 g/day. However, some CKD patients still have proteinuria after ACEI or ARB treatment (Igarashi et al., 2006; Slagman et al., 2011). Previous studies have suggested that the additive antiproteinuric and hypotensive effects of combined renin–angiotensin–aldosterone system (RAAS) blockade were superior to single RAAS blockade in CKD (Susantitaphong et al., 2013). Nonetheless, the use of ACEI in combination with ARB is not supported by all recent guidelines owing to concerns regarding adverse events such as renal dysfunction, hyperkalemia, and symptomatic hypotension in high-risk CKD patients (Esteras et al., 2015). Whether ACEI in combination with ARB or increasing the dose of ACEI or ARB is more effective in the treatment of CKD remains controversial. Therefore, the present meta-analysis of randomized controlled trials (RCTs) was designed to assess the efficacy and safety of ACEI in combination with ARB in patients with CKD based on the dose.

## Methods

### Data Sources and Searches

We searched PubMed, EMBASE, and Cochrane Library from inception to March 2020 to retrieve relevant articles. Two reviewers (Mingming Zhao and Rumeng Wang) independently screened the titles and abstracts of all electronic citations and full-text articles were retrieved for comprehensive review and independently rescreened. If a disagreement occurred between them, it was resolved by consulting with a third investigator (Yu Zhang). Medical Subject Headings and free-text terms were used in each database with the following relevant keywords: “diabetic nephropathy,” “hypertensive nephropathy,” “glomerular disease,” “proteinuria,” “renal insufficiency,” “kidney disease,” “chronic renal failure,” “chronic kidney disease,” “drug therapy combination,” “renin–angiotensin system,” “angiotensin-converting enzyme inhibitor,” and “angiotensin receptor blocker” (Supplementary Material S1).

### Study Selection

We included studies that met the following inclusion criteria: 1) patients (>18 years old) with CKD (KDIGO: CKD is defined as abnormalities of kidney structure or function, present for >3 months, with implications for health); 2) the intervention group received ACEI in combination with ARB (dual therapy), and the comparison group received ACEI or ARB (single therapy); 3) the outcomes involved albuminuria, proteinuria, GFR (creatinine clearance or estimated GFR), BP, hyperkalemia (>5.5 mmol/L or as defined in the individual studies), or hypotension (as defined in the individual studies); 4) randomized, controlled, crossover, or parallel trials; 5) the articles were published in English language.

### Data Extraction and Quality Assessment

Two reviewers (Mingming Zhao and Rumeng Wang) extracted data independently and disagreements were resolved by consulting with a third investigator (Yu Zhang). The following data were extracted from each of the published studies included in our review: the first author’s name, publication year, study design, intervention, dose of ACEI or ARB (low-dose: single dose compared with the same RAAS blockade in ACEI in combination with ARB group; high-dose: more than single dose compared with the same RAAS blockade in ACEI in combination with ARB group), sample size, percentage of men, mean age of subjects, duration of intervention, GFR, urine albumin or protein excretion rate, systolic blood pressure (SBP), diastolic blood pressure (DBP), mean arterial pressure, hyperkalemia, and hypotension. The methodological quality of the included studies was evaluated according to the recommendation of the Cochrane Handbook, including random sequence generation, allocation concealment, blinding of participants and personnel, blinding of outcome assessment, incomplete outcome data, selective reporting, and other bias. Marked 1 point when the risk was low.

### Data Synthesis and Analysis

The random effects model was used to calculate the effect sizes of eligible studies. For continuous outcomes, we calculated a weighted mean difference (WMD) or standard mean difference (SMD) with a 95% confidence interval (CI). For dichotomous outcomes, we estimated the relative risk (RR) with a 95% CI.

Heterogeneity of the included studies was described with the I^2^ index and the chi-square test. I^2^ ≥ 50% and *p* < 0.05 were used to indicate medium-to-high heterogeneity. We detected the potential sources of heterogeneity using meta-regression based on a priori selected study characteristics, including baseline of GFR, duration of intervention, mean age of subjects, and quality of included studies. Sensitivity analysis was performed to assess the robustness of the pooled results. The publication bias was evaluated using Begg’s test and Egger’s test. Statistical analysis was performed using Stata (version 15.1). The methodological quality of the included studies was assessed using RevMan5.3. We have registered the protocol for the present systematic review and meta-analysis, and the registration number in PROSPERO is CRD42020179398.

## Results

### Characteristics and Quality of the Studies

**FIGURE 1:**
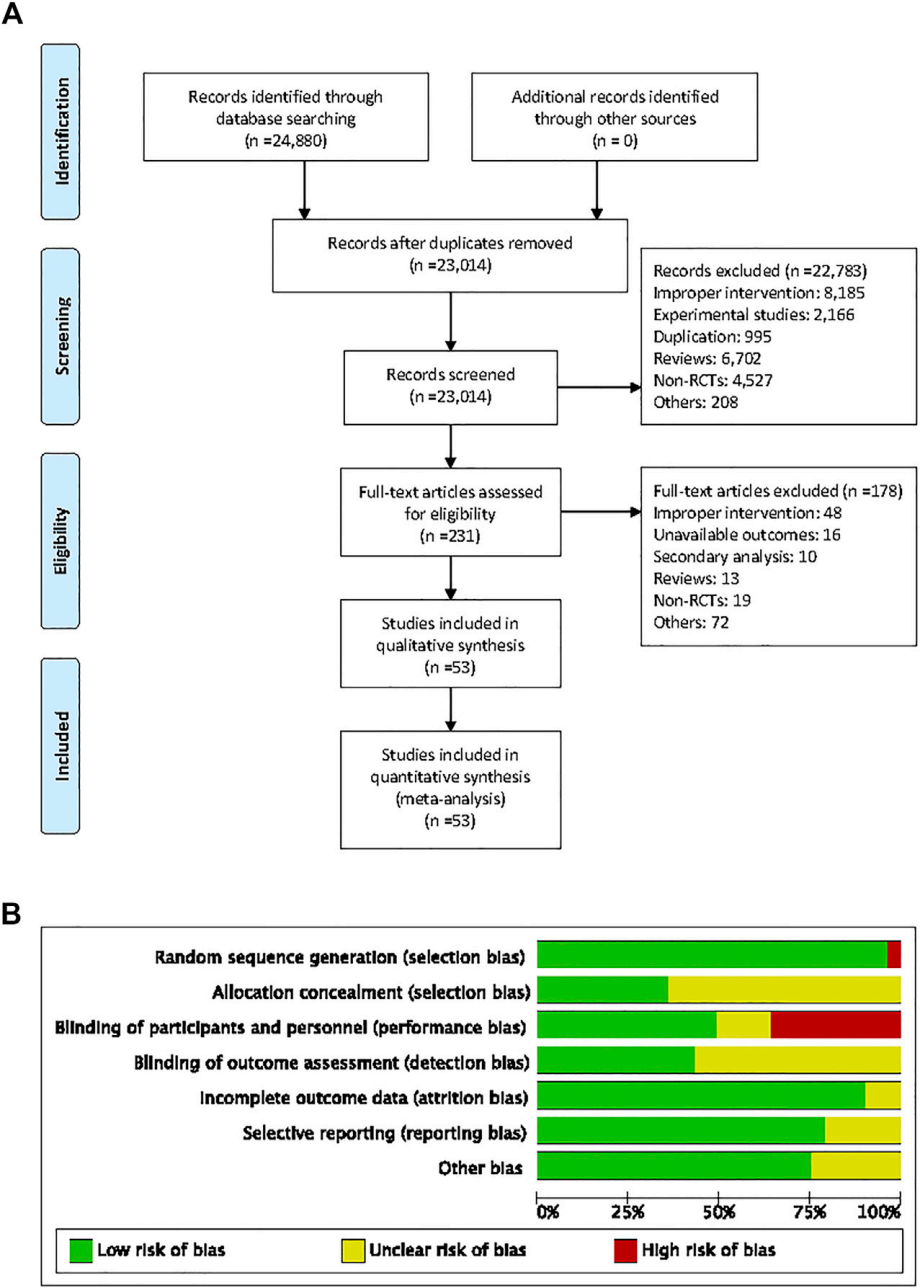
Flow diagram of searching for eligible studies and risk of bias summary. **(A)** Flow diagram of searching for eligible studies, **(B)** risk of bias summary.

A total of 24,880 studies (18,664 from PubMed, 4,034 from EMBASE, and 2,182 from the Cochrane Library) were identified, of which 53 studies met the inclusion criteria (Figure 1A).

The characteristics of the individual trials are presented in Table 1. Fifty-three studies with 6,375 patients consisted of 19 crossover and 34 parallel-arm RCTs. The sample size varied from 10 to 1,448. The mean age of the subjects of the trials ranged from 31 to 76 years, and the duration of intervention ranged from 1 to 60 months. Twenty-eight studies enrolled patients with GFR ≥60 mL/min or mL/min/1.73 m^2^ and eight studies enrolled patients with GFR <60 mL/min or mL/min/1.73 m^2^. Seventeen studies did not report the subjects’ baseline kidney function. Fourteen studies were of fair quality (score 1–3) and 39 were of good quality (score 4–7) (Figure 1B).

**TABLE 1:** Characteristics of randomized controlled trials included in this meta-analysis.

Studies	Design	Renin–angiotensin–aldosterone system blockade		N (T/C)	Male (%)		Age (Y)		Duration (months)	GFR (mL/min or mL/min/1.73 m^2^)	Albuminuria or proteinuria (g/g of creatinine or g/24 h)	SBP (mm Hg)	DBP (mm Hg)
		Dual therapy (mg/day)	Single therapy (mg/day)		T	C	T	C					
Saglimbene et al. (2018)	Parallel-arm	ACEI + ARB	ACEI	416/413	27.52	28.75	63.40	62.20	32.4	67.85	0.16^a^	137.90	80.50
		ACEI + ARB	ARB	416/414	27.52	28.54	63.40	62.60	32.4	69.10	0.17^a^	138.10	80.30
Schrier et al. (2014)	Parallel-arm	Lisinopril + telmisartan	Lisinopril + placebo	273/285	51.65	49.82	37.00	36.30	60	91.50	0.02^a^	NR	NR
Zwiech and Bruzda-Zweich. (2013)	Parallel-arm	Ramipril 5 mg + losartan 50 mg	Ramipril 10 mg	47/47	61.70	59.57	59.90	60.10	4	NR	NR	127.50	78.50
Nakamura et al. (2013)	Parallel-arm	Imidapril 5 mg + losartan 50 mg	Losartan 100 mg	14/14	71.43	64.29	61.70	61.40	12	87.75	0.25^a^	135.00	79.00
Fried et al. (2013)	Parallel-arm	Lisinopril 40 mg + losartan 100 mg	Losartan 100 mg + placebo	724/724	98.76	99.59	64.50	64.70	26.4	53.65	1.04^a^	136.95	72.65
Fernandez juarez et al. (2013)	Parallel-arm	Lisinopril 5 mg + irbesartan 75 mg	Lisinopril 10 mg	70/35	78.00	70.00	63.00	68.70	32	49.00	1.20	152.50	80.50
		Lisinopril 5 mg + irbesartan 75 mg	Irbesartan 150 mg	70/28	78.00	75.00	63.00	67.90	32	48.00	1.40	153.00	81.50
Titan et al. (2011)	Parallel-arm	Enalapril 40 mg + losartan 100 mg	Enalapril 40 mg + placebo	28/28	71.43	53.57	58.10	58.00	4	49.87	3.22	148.65	80.45
Slagman et al. (2011)	Crossover	Lisinopril 40 mg + valsartan 320 mg + low sodium	Lisinopril 40 mg + placebo + low sodium	52/52	82.69		51.50		1.5	70.50	1.63	131.00	76.25
		Lisinopril 40 mg + valsartan 320 mg + regular sodium	Lisinopril 40 mg + placebo + regular sodium	52/52	82.69		51.50		1.5	70.50	1.63	131.00	76.25
Meier et al. (2011)	Crossover	Losartan 100 mg + lisinopril 20 mg	Losartan 100 mg	20/20	50.00		53.00		2	67.00	6.39	138.50	83.50
		Losartan 100 mg + lisinopril 20 mg	Losartan 200 mg	20/20	50.00		53.00		2	67.00	6.39	138.50	83.50
Ohishi et al. (2010)	Parallel-arm	Imidapril 10 mg + valsartan 160 mg	Olmesartan 40 mg	18/19	86.49		64.00		4	41.05	1.70	106.00	86.00
Cice et al. (2010)	Parallel-arm	ACEI + telmisartan 80 mg	ACEI + placebo	165/167	53.33	54.49	62.70	62.80	36	NR	NR	125.40	81.00
Mehdi et al. (2009)	Parallel-arm	Lisinopril 80 mg + losartan 100 mg	Lisinopril 80 mg + placebo	26/27	50.00	44.44	52.30	49.30	12	68.90	0.91^a^	134.00	73.00
Krairittichai and Chaisuvannarat. (2009)	Parallel-arm	Enalapril 40 mg + telmisartan 80 mg	Enalapril 40 mg	40/40	53.75		55.67		6	46.33	2.31	140.46	75.47
Zhu et al. (2008)	Parallel-arm	Benazepril 10 mg + valsartan 80 mg	Benazepril 10 mg + placebo	27/28	55.56	57.14	56.00	55.00	3	NR	0.33^a^	153.50	95.50
		Benazepril 10 mg + valsartan 80 mg	Valsartan 80 mg + placebo	27/27	55.56	59.26	56.00	57.00	3	NR	0.33^a^	152.50	94.50
Mori-Takeyama et al. (2008)	Parallel-arm	Benazepril 2.5–10 mg + candesartan 4 mg	Candesartan 6–12 mg	39/38	56.41	63.16	36.90	37.80	36	94.95	1.35	134.15	82.60
Menne et al. (2008)	Parallel-arm	Lisinopril 20 mg + valsartan 320 mg	Lisinopril 40 mg	40/47	77.50	70.21	59.20	59.70	7.5	113.05	NR	151.70	90.35
		Lisinopril 20 mg + valsartan 320 mg	Valsartan 320 mg	40/42	77.50	66.67	59.20	57.00	7.5	119.75	NR	151.75	91.00
Knudsen et al. (2008)	Parallel-arm	Lisinopril 20 mg + candesartan 16 mg	Lisinopril 40 mg	25/26	72.00	80.77	56.00	57.00	12	117.50	NR	140.50	83.00
Ogawa et al. (2007)	Parallel-arm	Temocapril 2 mg + candesartan 4 mg	Temocapril 4 mg	37/34	48.65	47.06	61.80	60.90	24	NR	0.24^a^	154.00	91.15
		Temocapril 2 mg + candesartan 4 mg	Candesartan 8 mg	37/40	48.65	47.50	61.80	62.20	24	NR	0.24^a^	153.00	90.80
		Candesartan 4 mg + temocapril 2 mg	Temocapril 4 mg	35/34	48.57	47.06	62.50	60.90	24	NR	0.25^a^	151.50	90.20
		Candesartan 4 mg + temocapril 2 mg	Candesartan 8 mg	35/40	48.57	47.50	62.50	62.20	24	NR	0.25^a^	150.50	89.85
Nakamura et al. (2007)	Parallel-arm	Temocapril 2 mg + olmesartan 10 mg	Temocapril 2 mg	8/8	50.00	50.00	31.00	31.00	3	88.70	1.95	116.50	68.00
		Temocapril 2 mg + olmesartan 10 mg	Olmesartan 10 mg	8/8	50.00	62.50	31.00	34.00	3	88.50	1.95	117.50	69.00
Bakris et al. (2007)	Parallel-arm	Ramipril 10 mg + irbesartan 150–300 mg	Ramipril 10 mg + placebo	204/201	60.29	63.68	65.50	65.80	5	NR	NR	163.50	89.50
Abe et al. (2007)	Parallel-arm	ACEI + losartan 25 mg or 50 mg	ACEI	14/20	78.57	55.00	59.50	59.80	12	NR	1.35^a^	144.00	79.00
Song et al. (2006)	Crossover	Ramipril 5 mg + candesartan 8 mg	Ramipril 10 mg	21/21	52.38		49.00		4	40.60	4.10	133.00	81.00
		Ramipril 5 mg + candesartan 8 mg	Candesartan 16 mg	21/21	52.38		49.00		4	40.60	4.10	133.00	81.00
Sengul et al. (2006)	Parallel-arm	Lisinopril 20 mg + telmisartan 80 mg	Lisinopril 20 mg	47/48	38.30	35.42	57.00	57.20	7	95.15	0.16^a^	139.80	82.15
		Lisinopril 20 mg + telmisartan 80 mg	Telmisartan 80 mg	47/48	38.30	37.50	57.00	56.40	7	94.15	0.17^a^	139.95	83.30
		Telmisartan 80 mg + lisinopril 20 mg	Lisinopril 20 mg	49/48	40.82	35.42	56.90	57.20	7	94.70	0.17^a^	140.15	82.85
		Telmisartan 80 mg + lisinopril 20 mg	Telmisartan 80 mg	49/48	40.82	37.50	56.90	56.40	7	93.70	0.17^a^	140.30	84.00
Kanno et al. (2006)	Parallel-arm	ACEI + candesartan 2–12 mg	ACEI	45/45	40.00	40.00	60.30	59.50	36	NR	1.70	137.50	84.50
Igarashi et al. (2006)	Parallel-arm	Enalapril 5 mg + losartan 50 mg	Enalapril 10 mg	13/13	76.92	61.54	63.50	63.90	3	75.55	1.81	148.70	80.45
Horita et al. (2006)	Parallel-arm	Temocapril 1 mg + losartan 12.5 mg	Temocapril 1 mg	13/14	53.85	57.14	38.00	43.00	12	92.55	0.70	118.00	73.00
		Temocapril 1 mg + losartan 12.5 mg	Losartan 12.5 mg	13/16	53.85	56.25	38.00	42.00	12	91.65	0.82	123.50	78.00
Chrysostomou et al. (2006)	Parallel-arm	Ramipril 5 mg + irbesartan 150 mg	Ramipril 5 mg + placebo	10/10	80.00	70.00	56.30	59.20	3	74.80	2.55	132.50	79.50
Atmaca and Gedik. (2006)	Parallel-arm	Lisinopril 10 mg + losartan 50 mg	Lisinopril 10 mg	8/9	37.50	44.44	55.10	55.10	12	NR	0.07^a^	120.00	78.30
		Lisinopril 10 mg + losartan 50 mg	Losartan 50 mg	8/9	37.50	44.44	55.10	55.10	12	NR	0.07^a^	120.00	78.85
Scaglione et al. (2005)	Parallel-arm	Ramipril 5 mg + losartan 50 mg	Ramipril 5 mg + placebo	17/17	47.06	47.06	58.00	54.00	6	71.50	0.45^a^	160.50	95.50
		Ramipril 5 mg + losartan 50 mg	Losartan 50 mg + placebo	17/17	47.06	47.06	58.88	56.00	6	70.00	0.41^a^	162.50	93.00
Matos et al. (2005)	Crossover	Perindopril 8 mg + irbesartan 300 mg	Perindopril 8 mg	20/20	25.00		54.74		4	67.00	0.90	154.00	86.00
		Perindopril 8 mg + irbesartan 300 mg	Irbesartan 300 mg	20/20	25.00		54.74		4	67.00	0.97	153.50	86.00
Esnault et al. (2005)	Crossover	Ramipril 5 mg + valsartan 80 mg	Ramipril 10 mg	18/18	66.67		49.30		1	NR	3.71	149.06	83.00
		Ramipril 5 mg + valsartan 80 mg	Valsartan 160 mg	18/18	66.67		49.30		1	NR	3.71	149.06	83.00
Rutkowski et al. (2004)	Crossover	Benazepril 5 mg + losartan 25 mg	Benazepril 10 mg	24/24	50.00		35.46		4	85.72	2.13	139.52	90.73
		Benazepril 5 mg + losartan 25 mg	Losartan 50 mg	24/24	50.00		35.46		4	85.72	2.13	139.52	90.73
Renke et al. (2004)	Parallel-arm	Enalapril 10 mg + losartan 25 mg	Enalapril 10 mg	16/18	68.75	66.67	37.70	43.40	9	94.35	2.93	137.00	89.30
		Enalapril 10 mg + losartan 25 mg	Losartan 25 mg	16/18	68.75	38.89	37.70	40.40	9	93.65	2.71	138.55	89.50
Nakao et al. (2004)	Parallel-arm	Trandolapril 3 mg + losartan 100 mg	Trandolapril 3 mg	31/31	58.06	54.84	43.20	43.30	36	46.35	1.95	138.00	81.00
		Trandolapril 3 mg + losartan 100 mg	Losartan 100 mg	31/30	58.06	56.67	42.90	43.40	36	45.90	1.90	137.00	80.50
Morgan et al. (2004)	Crossover	Lisinopril 20 mg + candesartan 16 mg	Lisinopril 20 mg	23/23	95.65		75.60		1	77.00	NR	142.00	79.80
		Lisinopril 20 mg + candesartan 16 mg	Lisinopril 40 mg	23/22	95.65		75.60		1	77.00	NR	142.00	79.80
		Lisinopril 20 mg + candesartan 16 mg	Candesartan 16 mg	23/23	95.65		75.60		1	77.00	NR	142.00	79.80
		Lisinopril 20 mg + candesartan 16 mg	Candesartan 32 mg	23/23	95.65		75.60		1	77.00	NR	142.00	79.80
Horita et al. (2004)	Parallel-arm	Temocapril 1 mg + losartan 12.5 mg	Temocapril 1 mg	11/10	45.45	40.00	39.60	39.60	6	92.00	0.74	121.00	75.50
		Temocapril 1 mg + losartan 12.5 mg	Losartan 12.5 mg	11/10	45.45	50.00	39.60	42.70	6	89.90	0.78	122.50	76.50
Song et al. (2003)	Crossover	Ramipril 5–7.5 mg + candesartan 4–8 mg	Ramipril 5–7.5 mg + placebo	14/14	42.86		31.00		4	60.30	4.00	91.20^b^	
		Ramipril 5–7.5 mg + candesartan 4–8 mg	Ramipril 5–7.5 mg + placebo	18/18	38.89		42.00		4	59.40	4.10	92.30^b^	
Segura et al. (2003)	Parallel-arm	Benazepril 10–20 mg + valsartan 160 mg	Benazepril10–20 mg	12/12	83.33	66.67	47.90	49.80	6	70.00	3.95	151.50	90.50
		Benazepril 10–20 mg + valsartan 160 mg	Valsartan 160 mg	12/12	83.33	66.67	47.90	49.70	6	71.00	4.35	150.50	88.00
Rossing et al. (2003)	Crossover	ACEI + candesartan 16 mg	ACEI + placebo	20/20	85.00		62.00		2	NR	>0.30^a^	NR	NR
Kim et al. (2003)	Crossover	Ramipril 5 mg + candesartan 4 mg	Ramipril 5 mg + placebo	41/41	46.34		34.00		3	61.20	4.00	93.00^b^	
Jacobsen et al. (2003a)	Crossover	Enalapril 40 mg + irbesartan 300 mg	Enalapril 40 mg + placebo	24/24	70.83		42.00		2	NR	>0.30^a^	NR	NR
Jacobsen et al. (2003b)	Crossover	Benazepril 20 mg + valsartan 80 mg	Benazepril 20 mg	18/18	72.22		43.00		2	NR	0.67^a^	141.00	81.00
		Benazepril 20 mg + valsartan 80 mg	Valsartan 80 mg	18/18	72.22		43.00		2	NR	0.67^a^	141.00	81.00
Campbell et al. (2003)	Crossover	Benazepril 10 mg + valsartan 80 mg	Benazepril 20 mg	24/24	95.83		48.90		2	69.14	3.28	140.00	91.00
		Benazepril 10 mg + valsartan 80 mg	Valsartan 160 mg	24/24	95.83		48.90		2	69.14	3.28	140.00	91.00
Rossing et al. (2002)	Crossover	ACEI + candesartan 8 mg	ACEI + placebo	18/18	76.47		58.00		2	NR	1.78^a^	159.00	85.00
Nakamura et al. (2002)	Parallel-arm	Trandolapril 2 mg + candesartan 8 mg	Trandolapril 2 mg	15/15	73.33	66.67	57.80	57.00	18	102.60	NR	123.00	75.00
		Trandolapril 2 mg + candesartan 8 mg	Candesartan 8 mg	15/15	73.33	60.00	57.80	56.50	18	103.70	NR	122.00	74.00
Luño et al. (2002)	Parallel-arm	Lisinopril 20 mg + candesartan 16 mg	Lisinopril 40 mg	16/14	56.25	85.71	42.00	50.00	6	90.00	3.70	135.00	82.00
		Lisinopril 20 mg + candesartan 16 mg	Candesartan 32 mg	16/15	56.25	66.67	42.00	45.00	6	100.00	3.90	134.00	82.00
Kincaid-smith et al. (2002)	Crossover	ACEI + candesartan 8 mg	ACEI	60/60	NR	NR	NR	NR	3	NR	2.30	136.50	83.00
Jacobsen et al. (2002)	Crossover	ACEI + irbesartan 300 mg	ACEI + placebo	21/21	80.95		45.00		2	NR	1.87^a^	156.00	87.00
Ferrari et al. (2002)	Crossover	Fosinopril 20 mg + irbesartan 150 mg	Fosinopril 20 mg	10/10	70.00		48.00		1.5	77.00	7.90	143.50	91.00
		Fosinopril 20 mg + irbesartan 150 mg	Irbesartan 150 mg	10/10	70.00		48.00		1.5	77.00	7.90	143.50	91.00
Bergeret al. (2002)	Crossover	ACEI + candesartan 8 mg	ACEI + placebo	12/12	50.00		52.00		2	65.00	2.00	128.50	79.00
Tütüncü et al. (2001)	Parallel-arm	Enalapril 5 mg + losartan 50 mg	Enalapril 5 mg	10/12	NR		57.70	51.40	12	NR	0.09^a^	117.50	75.00
		Enalapril 5 mg + losartan 50 mg	Losartan 50 mg	10/12	NR		57.70	58.10	12	NR	0.10^a^	117.50	77.50
Agarwal. (2001)	Crossover	Lisinopril 40 mg + losartan 50 mg	Lisinopril 40 mg + placebo	16/16	NR		53.00		1	66.00	3.58	156.00	88.00
Ruilope et al. (2000)	Parallel-arm	Benazepril 5 or 10 mg + valsartan 80 mg	Valsartan 160 mg	42/22	70.00	73.00	56.90	57.30	1	NR	1.81	156.50	93.50
		Benazepril 5 or 10 mg + valsartan 160 mg	Valsartan 160 mg	44/22	66.00	73.00	57.60	57.30	1	NR	1.77	157.50	94.50

**Abbreviations:** N, Number of patients; T, treatment group; C, control group; Y, year; sCr, serum creatinine; GFR, glomerular filtration rate; SBP, systolic blood pressure; DBP, diastolic blood pressure; ACEI, angiotensin-converting enzyme inhibitor; ARB, angiotensin-receptor blocker; NR, not reported.

a Value represents urinary albumin excretion.

b Mean arterial pressure.

### Efficacy and Safety of ACEI in Combination with ARB vs. Low-Dose ACEI or ARB

**FIGURE 2:**
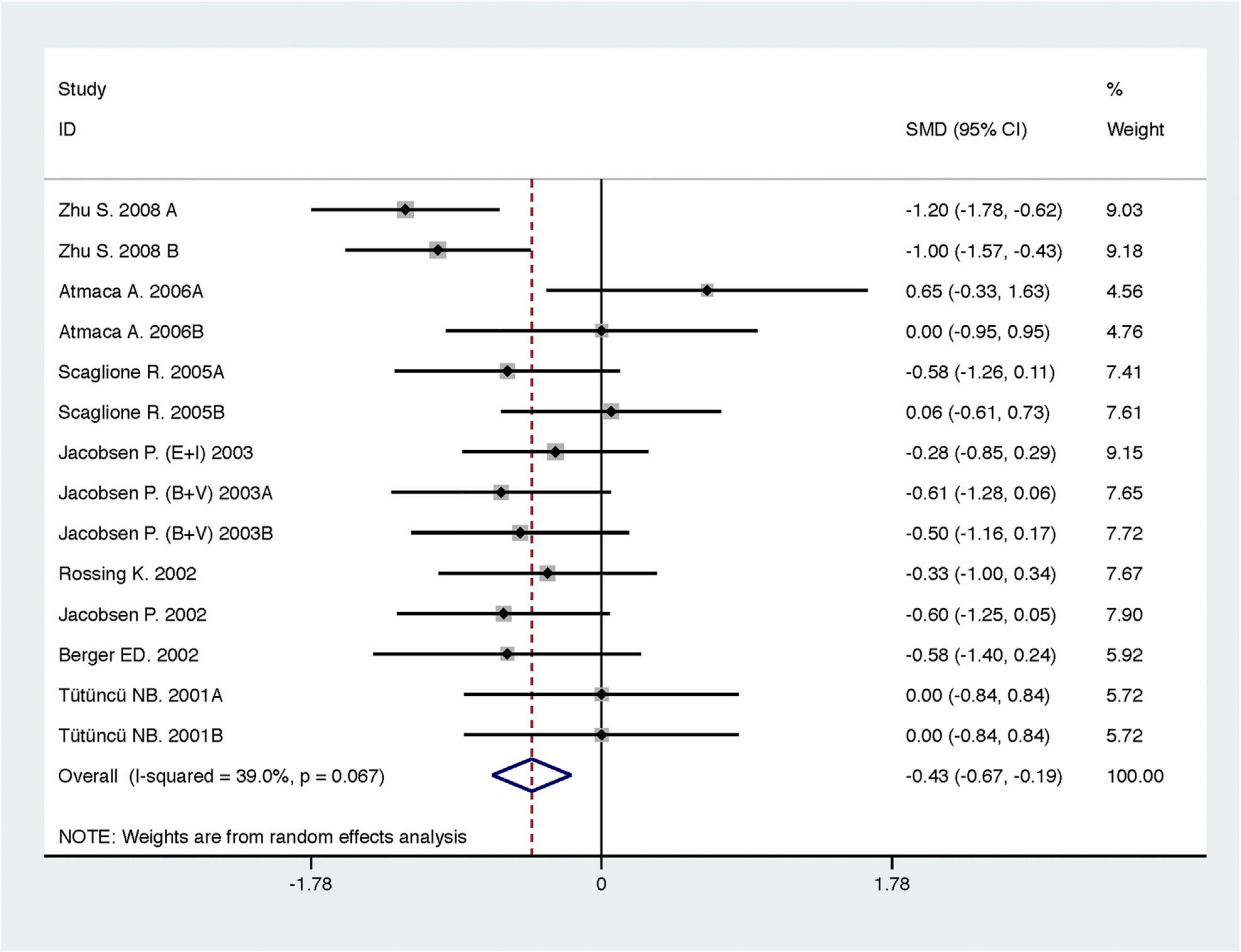
Comparison of ACEI in combination with ARB vs. low-dose ACEI or ARB for urine albumin excretion (g/g of creatinine or g/24 h).

**FIGURE 3:**
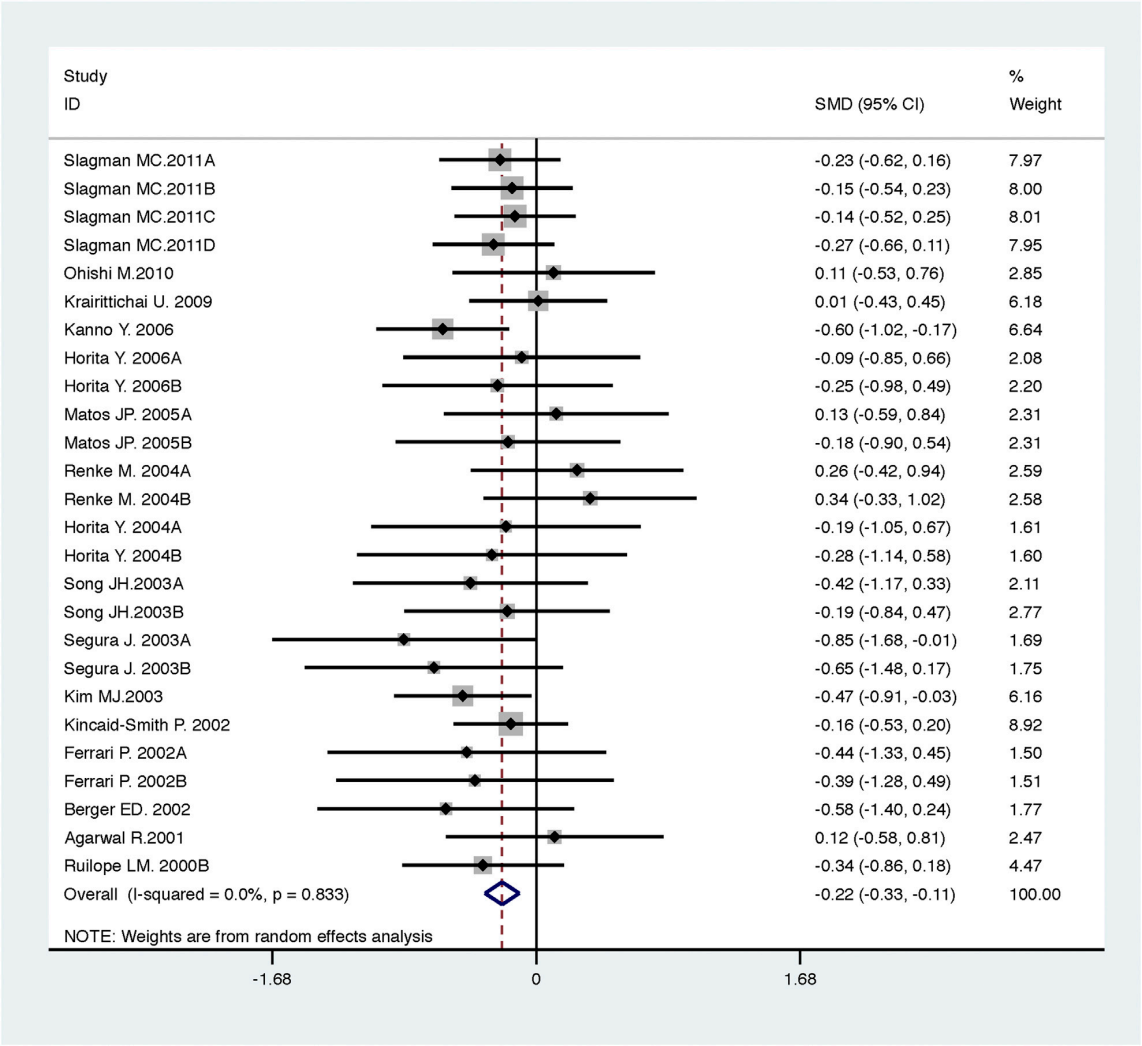
Comparison of ACEI in combination with ARB vs. low-dose ACEI or ARB for urine protein excretion (g/g of creatinine or g/24 h).

**FIGURE 4:**
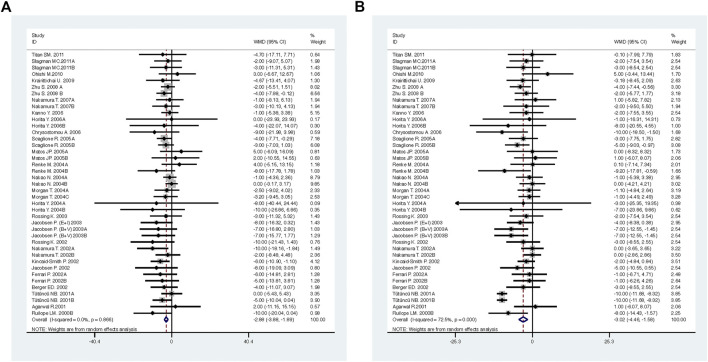
Comparison of ACEI in combination with ARB vs. low-dose ACEI or ARB for blood pressure. **(A)** systolic blood pressure (mmHg), **(B)** diastolic blood pressure (mmHg).

**FIGURE 5:**
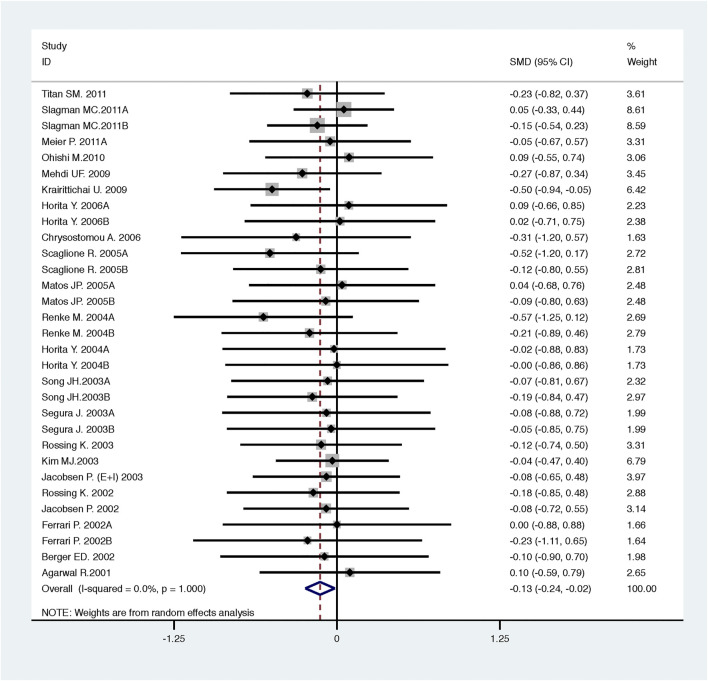
Comparison of ACEI in combination with ARB vs. low-dose ACEI or ARB for glomerular filtration rate (mL/min or mL/min/1.73m^2^).

**FIGURE 6:**
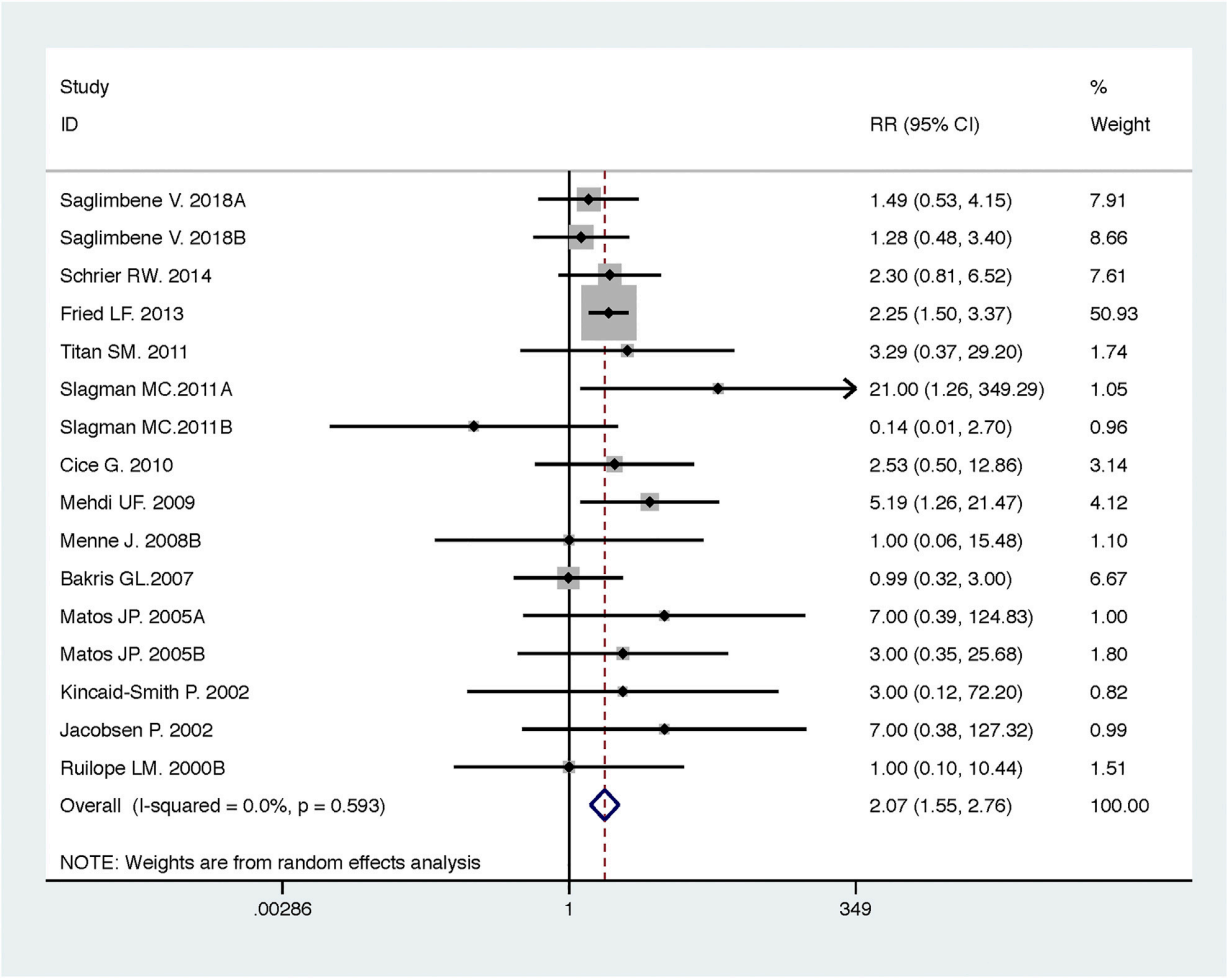
Comparison of ACEI in combination with ARB vs. low-dose ACEI or ARB for development of hyperkalemia.

**FIGURE 7:**
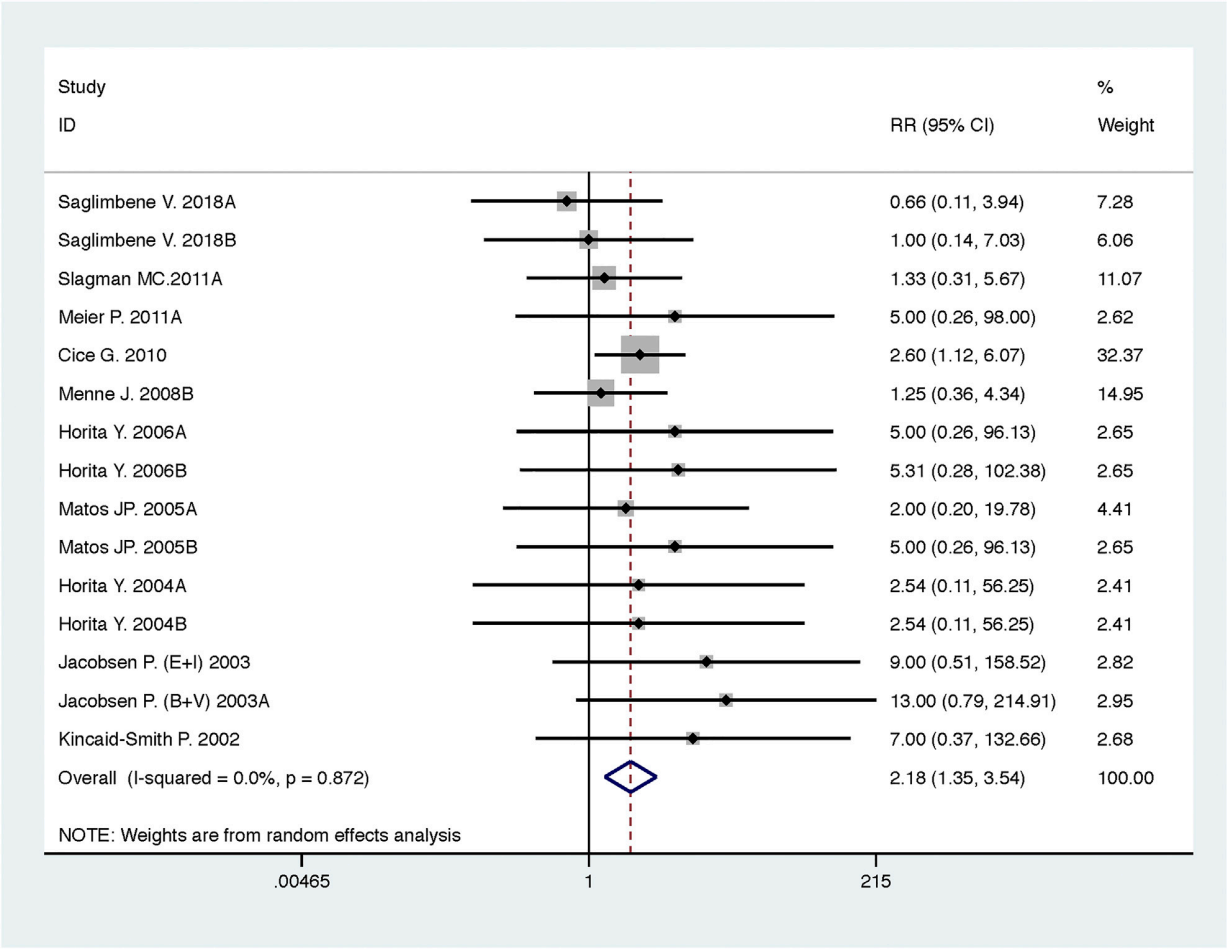
Comparison of ACEI in combination with ARB vs. low-dose ACEI or ARB for development of hypotension.

Compared with low-dose ACEI or ARB, ACEI in combination with ARB significantly reduced urine albumin excretion (SMD, −0.43; 95% CI, −0.67 to −0.19; *p* = 0.001), urine protein excretion (SMD, −0.22; 95% CI, −0.33 to −0.11; *p* < 0.001), and BP (SBP: WMD, −2.89; 95% CI, −3.88 to −1.89; *p* < 0.001; DBP: WMD, −3.02; 95% CI, −4.46 to −1.58; *p* < 0.001) (Table 2; Figures 2–4). However, dual therapy was associated with decreased GFR (SMD, −0.13; 95% CI, −0.24 to −0.02; *p* = 0.02), increased rates of hyperkalemia (RR, 2.07; 95% CI, 1.55 to 2.76; *p* < 0.001) and hypotension (RR, 2.19; 95% CI, 1.35 to 3.54; *p* = 0.001) compared with low-dose ACEI or ARB (Table 2; Figures 5–7).

**TABLE 2:** Summary effect of ACEI in combination with ARB vs. ACEI or ARB.

Outcome	No. study arms	No. participants	Random-effects model		Assessment of heterogeneity		Publication bias (*p*-value)	
			95% CI	*p*-value	I^2^ (%)	*p*-value	Begg’s test	Egger’s test
ACEI in combination with ARB vs. low-dose ACEI or ARB								
Urine albumin excretion (g/g of creatinine or g/24 h)	14	472	SMD: −0.43 (−0.67, −0.19)	0.001	39.0	0.07	0.01	0.01
Urine protein excretion (g/g of creatinine or g/24 h)	26	1,321	SMD: −0.22 (−0.33, −0.11)	<0.001	0.0	0.83	0.09	0.92
Glomerular filtration rate (mL/min or mL/min/1.73m^2^)	31	1,216	SMD: −0.13 (−0.24, −0.02)	0.02	0.0	1.00	0.87	0.79
Systolic blood pressure (mmHg)	41	1,727	WMD: −2.89 (−3.88, −1.89)	<0.001	0.0	0.87	0.17	0.06
Diastolic blood pressure (mmHg)	41	1,727	WMD: −3.02 (−4.46, −1.58)	<0.001	72.5	<0.001	0.07	<0.001
Development of hyperkalemia	16	5,079	RR: 2.07 (1.55, 2.76)	<0.001	0.0	0.59	0.50	0.81
Development of hypotension	15	2,590	RR: 2.19 (1.35, 3.54)	0.001	0.0	0.87	0.37	0.11
ACEI in combination with ARB vs. high-dose ACEI or ARB								
Urine albumin excretion (g/g of creatinine or g/24 h)	6	446	SMD: −0.84 (−1.26, −0.43)	<0.001	75.4	0.001	0.71	0.02
Urine protein excretion (g/g of creatinine or g/24 h)	17	851	SMD: −0.24 (−0.39, −0.09)	0.002	11.4	0.32	0.04	0.02
Glomerular filtration rate (mL/min or mL/min/1.73m^2^)	16	866	SMD: 0.02 (−0.12, 0.15)	0.78	0.0	0.80	0.30	0.98
Systolic blood pressure (mmHg)	25	1,369	WMD: −0.19 (−1.28, 0.91)	0.74	0.0	0.53	0.94	0.01
Diastolic blood pressure (mmHg)	25	1,369	WMD: −0.57 (−1.36, 0.22)	0.16	0.0	0.91	0.91	0.82
Development of hyperkalemia	6	441	RR: 0.94 (0.65, 1.37)	0.76	0.0	0.71	0.26	0.02
Development of hypotension	4	214	RR: 3.95 (1.13, 13.84)	0.03	0.0	0.93	0.73	0.82

### Efficacy and Safety of ACEI in Combination with ARB vs. High-Dose ACEI or ARB

**FIGURE 8:**
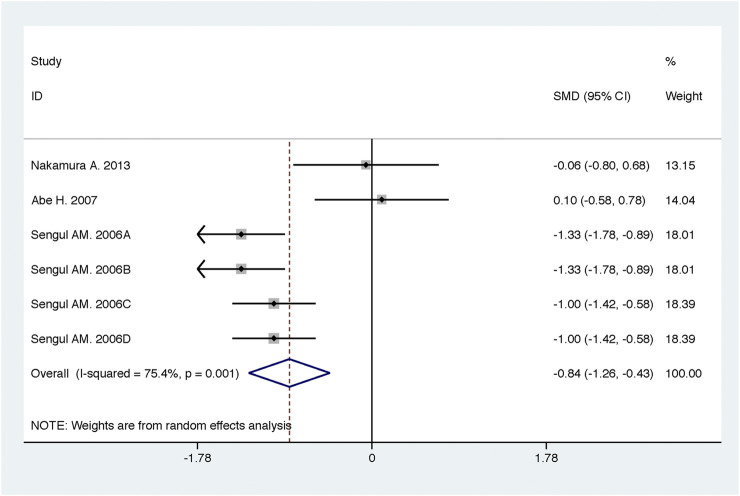
Comparison of ACEI in combination with ARB vs. high-dose ACEI or ARB for urine albumin excretion (g/g of creatinine or g/24 h).

**FIGURE 9:**
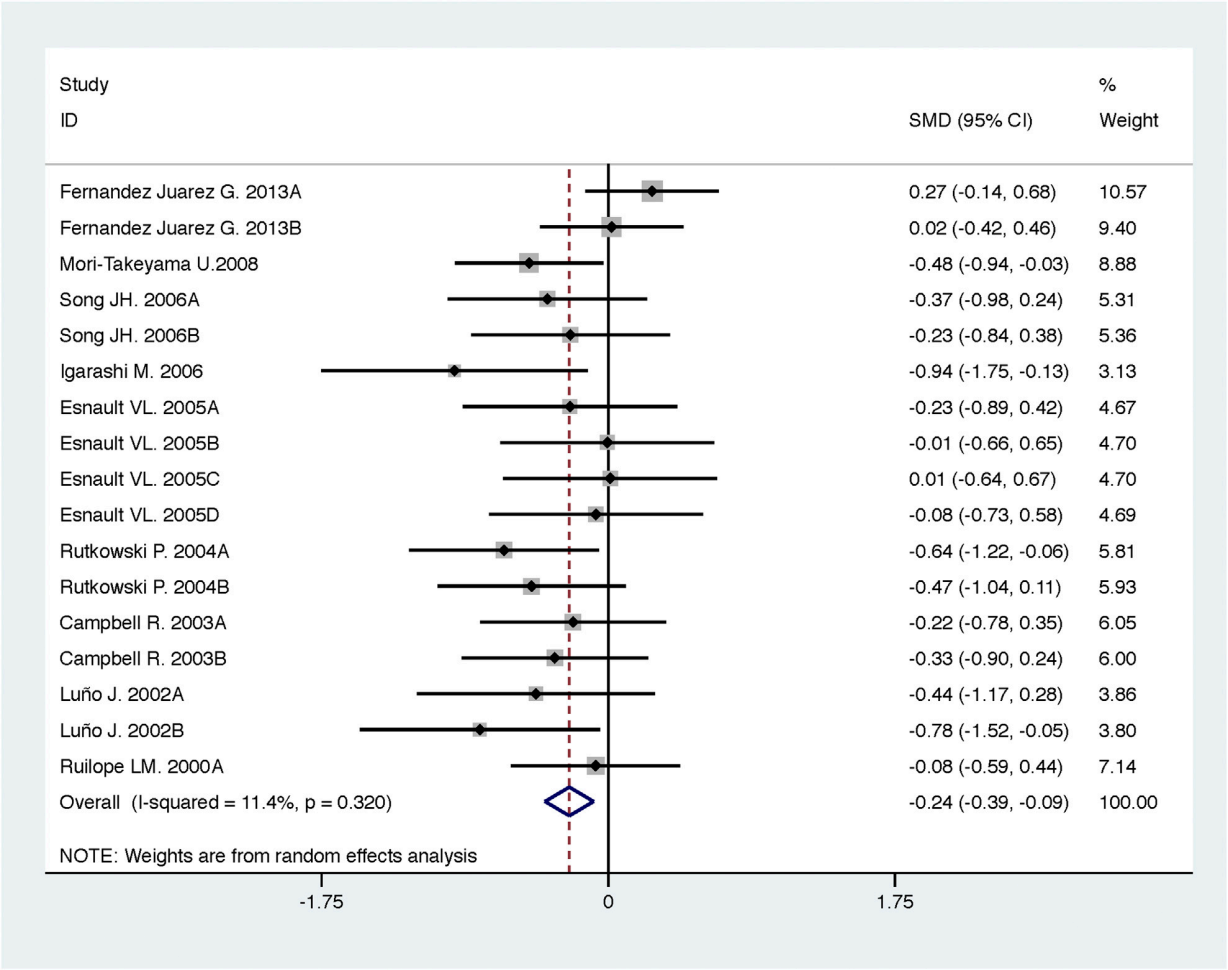
Comparison of ACEI in combination with ARB vs. high-dose ACEI or ARB for urine protein excretion (g/g of creatinine or g/24 h).

**FIGURE 10:**
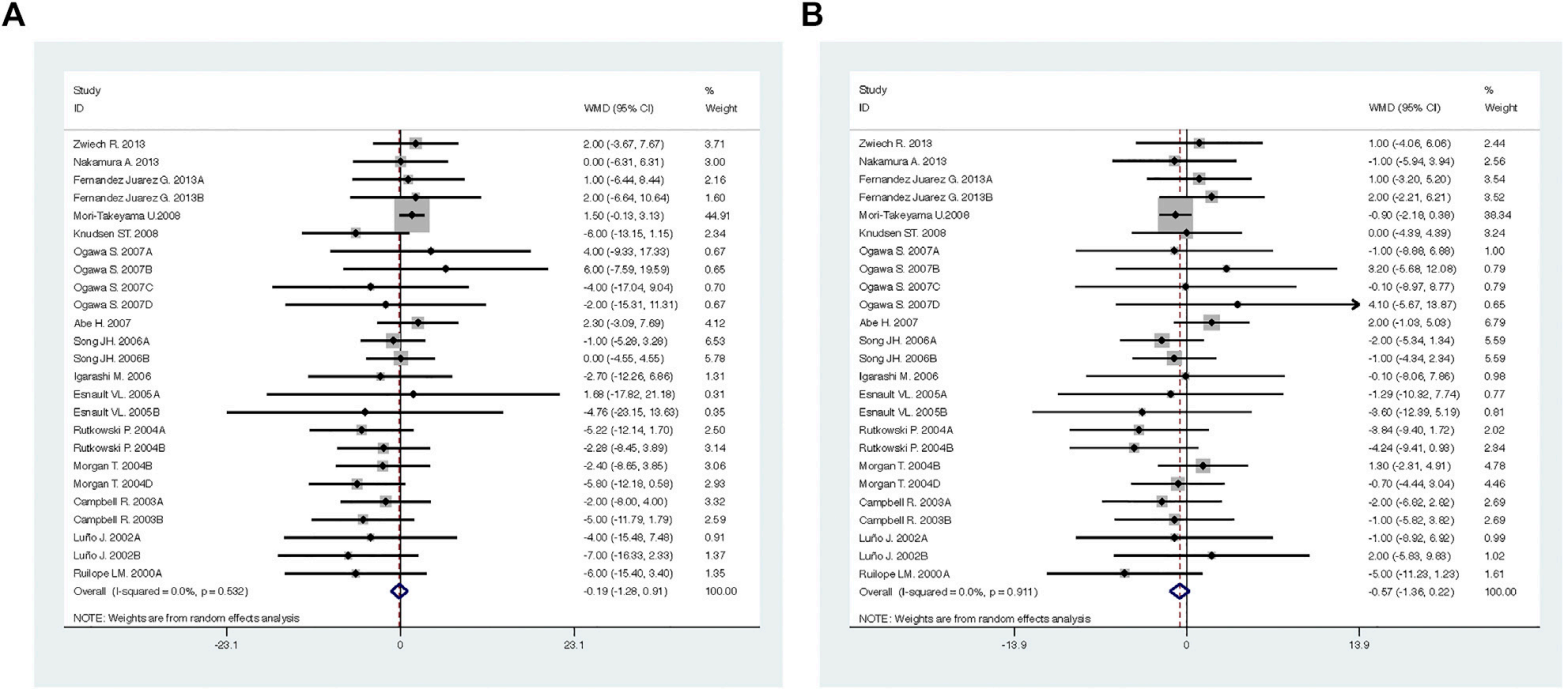
Comparison of ACEI in combination with ARB vs. high-dose ACEI or ARB for blood pressure. **(A)** systolic blood pressure (mmHg), **(B)** diastolic blood pressure (mmHg).

**FIGURE 11:**
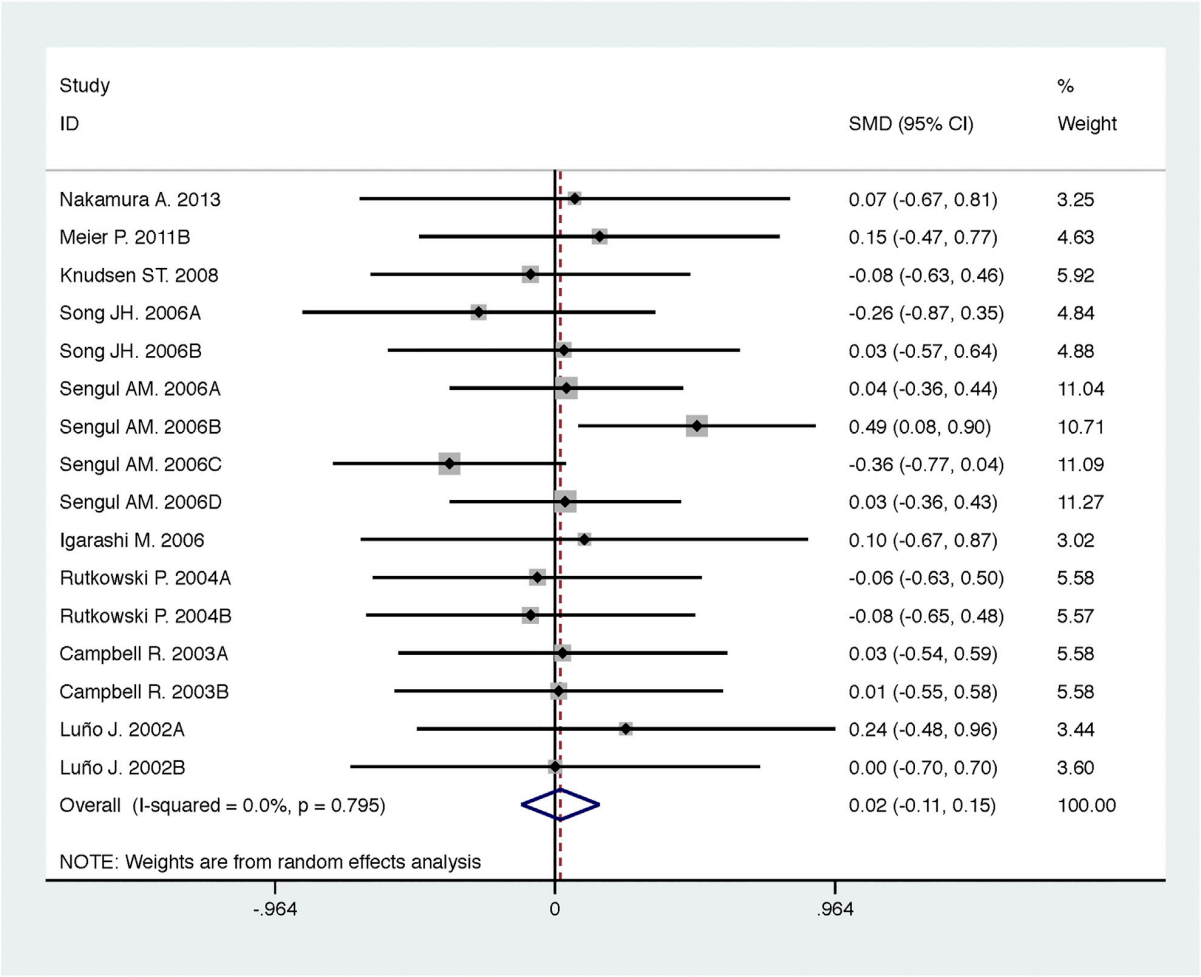
Comparison of ACEI in combination with ARB vs. high-dose ACEI or ARB for glomerular filtration rate (mL/min or mL/min/1.73m^2^).

**FIGURE 12:**
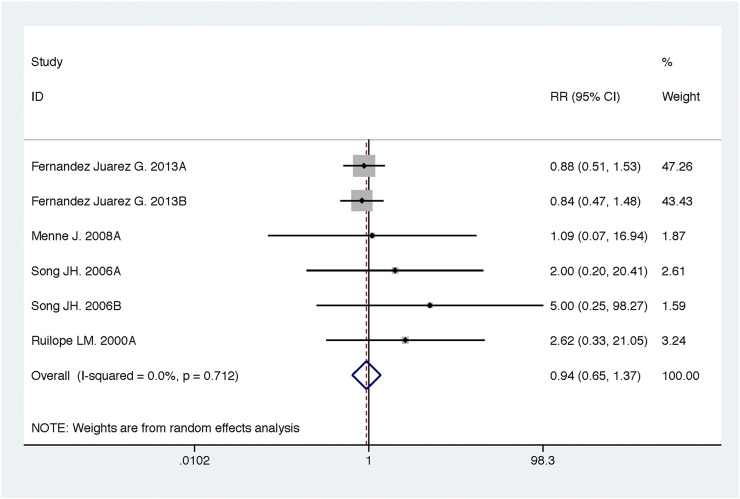
Comparison of ACEI in combination with ARB vs. high-dose ACEI or ARB for development of hyperkalemia.

**FIGURE 13:**
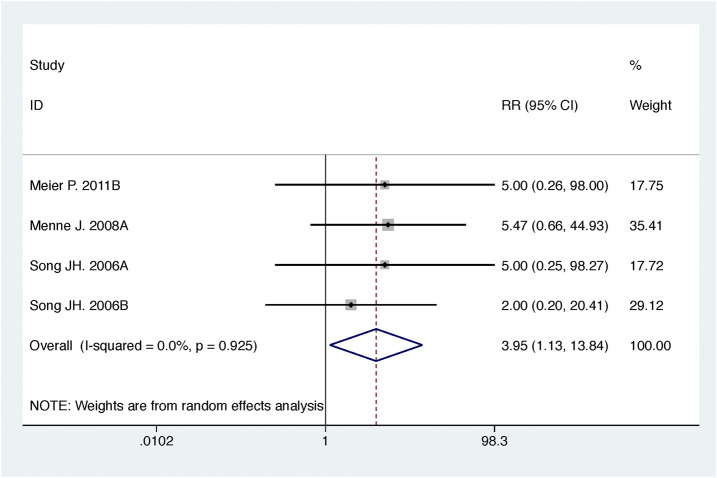
Comparison of ACEI in combination with ARB vs. high-dose ACEI or ARB for development of hypotension.

Compared with high-dose ACEI or ARB, ACEI in combination with ARB significantly reduced urine albumin excretion (SMD, −0.84; 95% CI, −1.26 to −0.43; *p* < 0.001) and urine protein excretion (SMD, −0.24; 95% CI, −0.39 to −0.09; *p* = 0.002) (Table 2; Figures 8, 9). The combination did not decrease SBP (WMD, −0.19; 95% CI, −1.28 to 0.91; *p* = 0.74) and DBP (WMD, −0.57; 95% CI, −1.36 to 0.22; *p* = 0.16) (Table 2; Figure 10). ACEI in combination with ARB was not associated with decreased GFR (SMD, 0.02; 95% CI, −0.12 to 0.15; *p* = 0.78) and an increased rate of hyperkalemia (RR, 0.94; 95% CI, 0.65 to 1.37; *p* = 0.76) compared with high-dose ACEI or ARB (Table 2; Figures 11, 12). However, dual therapy was associated with an increased rate of hypotension (RR, 3.95; 95% CI, 1.13 to 13.84; *p* = 0.03) (Table 2; Figure 13).

### Sensitivity Analysis and Meta-Regression

To ensure reliability of the present meta-analysis, we evaluated the robustness of the results (Table 2) using sensitivity analysis, which indicated that the results of the meta-analysis were robust.

Significant heterogeneities were observed for DBP and urine albumin excretion (Table 2). We detected the potential sources of heterogeneity using meta-regression based on a priori selected study characteristics, including the mean age of subjects, duration of intervention, baseline of GFR, and quality of included studies.

A significant heterogeneity was observed for the outcome of urine albumin excretion (Table 2, summary effect of ACEI in combination with ARB vs. high-dose ACEI or ARB, I^2^ = 75.4%, *p* = 0.001), which was dependent on the mean age of subjects (exp, 1.30; 95% CI, 1.04 to 1.63; adjusted *R* ^2^ = 89.09%; *p* = 0.03) and duration of intervention (exp, 1.27; 95% CI, 1.09 to 1.48; adjusted *R* ^2^ = 100.00%; *p* = 0.01). Using meta-regression, it was found that the heterogeneity of DBP (Table 2, summary effect of ACEI in combination with ARB vs. low-dose ACEI or ARB) was not associated with a priori selected study characteristics.

### Publication Bias

Begg’s test and Egger’s test were used to evaluate publication bias based on the key outcomes of the trials included in the meta-analysis. The results suggested less susceptibility to publication bias, except for urine albumin excretion and urine protein excretion (Table 2).

## Discussion

In the present meta-analysis of 53 RCTs encompassing 6,375 participants, we aimed to compare the efficacy and safety of ACEI in combination with ARB vs. low-dose and high-dose ACEI or ARB. We demonstrated that ACEI in combination with ARB was superior to low-dose ACEI or ARB in reducing urine albumin excretion, urine protein excretion, and BP, including SBP and DBP. However, the combination was associated with a decreased GFR and increased rates of hyperkalemia and hypotension. ACEI in combination with ARB was more effective in reducing urine albumin excretion and urine protein excretion than high-dose ACEI or ARB, without decreased GFR and increased rate of hyperkalemia. Nonetheless, the combination did not decrease the BP and increased the rate of hypotension compared with the high-dose ACEI or ARB.

Proteinuria and hypertension are risk factors for CKD progression (Liu and Lv, 2019; Nagai et al., 2019). Proteinuria is also an independent predictor of all-cause mortality. A combination of severely decreased GFR and proteinuria further increases the risk of all-cause mortality (Wu et al., 2018). For CKD patients with proteinuria, the updated hypertension guidelines recommend a BP goal of <130/80 mmHg (Hamrahian, 2017). More-intensive BP control is associated with a reduced risk of all-cause mortality compared with less-intensive BP goals in this high-risk population (Juraschek and Appel, 2018). Nevertheless, proportions with uncontrolled BP were greater in those with CKD than in those without CKD, and multiple medications and ACEI/ARB were associated with less uncontrolled BP (Plantinga et al., 2009). It should be emphasized that to lower albuminuria and achieve BP goals, moderate to high doses of ACEI or ARB are often required. However, ACEI or ARB may only reduce proteinuria by up to 40–50% in a dose-dependent manner, particularly if the patient complies with dietary salt restriction (Nakamura et al., 2000). This leads to a recommendation to use a more complete RAAS blockade to maximize kidney protection and improve outcomes. In order to study the effect of dose on ACEI in combination with ARB, we defined low-dose and high-dose as relative values. Compared with the same RAAS blocker in ACEI in combination with ARB group, a low-dose was defined as single dose, and the high-dose was defined as greater than single dose. According to our meta-analysis, ACEI in combination with ARB was superior to low-dose and high-dose ACEI or ARB in reducing urine albumin excretion and urine protein excretion. It is more effective to use ACEI in combination with ARB than to increase the dose of ACEI or ARB.

Although experimental and clinical studies have demonstrated that dual RAAS blockade therapy is more effective in reducing proteinuria and preventing structural lesions than either drug alone (Susantitaphong et al., 2013; Zhang et al., 2017), it is associated with higher incidences of adverse effects than monotherapy. The key safety issues associated with ACEI in combination with ARB are hypotension, which may lead to syncope, and impaired kidney function, which may lead to hyperkalemia (Oktaviono and Kusumawardhani, 2020). In this meta-analysis, although ACEI in combination with ARB was associated with a decrease in GFR and increased incidences of hyperkalemia and hypotension relative to low-dose ACEI or ARB, dual therapy did not decrease GFR nor increase the incidence of hyperkalemia compared with high-dose ACEI or ARB. Except for hypotension, the safety of ACEI in combination with ARB was equivalent to that of high-dose ACEI or ARB, and hypotension in some patients is temporary and mild (Song et al., 2006; Meier et al., 2011).

In recent years, the use of ACEI in combination with ARB has raised controversies, and no systemic review and meta-analysis have analyzed the efficacy and safety of the use of ACEI in combination with ARB in patients with CKD. This meta-analysis evaluated the effect of ACEI in combination with ARB on kidney-related endpoints, BP, and adverse events based on the dose. However, there are certain limitations to this study. First, only a few RCTs have evaluated the efficacy and safety of ACEI in combination with ARB vs. high-dose ACEI or ARB. More large-scale studies are needed to further clarify the application prospect of ACEI in combination with ARB in CKD. Second, some of the studies included in the present analysis were of a fair quality. Third, the included studies were heterogeneous; we performed sensitivity analysis and meta-regression to warrant the reliability of the present meta-analysis. Fourth, most of the included studies were aimed at CKD patients with a normal GFR or only a mildly reduced GFR. There are few with moderately reduced renal function and none with severely reduced renal function. The results of this meta-analysis are only applicable to CKD patients with a fairly maintained kidney function.

## Conclusion

In conclusion, ACEI in combination with ARB is superior to low-dose and high-dose ACEI or ARB in reducing urine albumin excretion and urine protein excretion. Although ACEI in combination with ARB is associated with a decreased GFR and increased rates of hyperkalemia and hypotension compared with low-dose ACEI or ARB, the combination is more effective than high-dose ACEI or ARB without decreasing GFR and increasing the incidence of hyperkalemia. Despite the risk of hypotension, ACEI in combination with ARB is a better choice for CKD patients who need to increase the dose of ACEI or ARB. The results of this meta-analysis are only applicable to CKD patients with a fairly maintained kidney function.

## Data Availability

The original contributions presented in the study are included in the article/Supplementary Material; further inquiries can be directed to the corresponding authors.
