# Supplementary material for: Efficacy and Safety of Angiotensin-Converting Enzyme Inhibitor in Combination with Angiotensin-Receptor Blocker in Chronic Kidney Disease Based on Dose: A Systematic Review and Meta-Analysis
Source: Front Pharmacol. 2021 May 6;12:638611. doi: 10.3389/fphar.2021.638611 (PMC8134749; doi:10.3389/fphar.2021.638611)
Supplement: Supplementary file 2 [file DataSheet1.PDF]

Databases	Search	Search Strings	Hits (2020.3.16)
PubMed	#1	((((((((diabetic nephropathy[MeSH Terms]) OR hypertensive nephropathy[MeSH Terms]) OR glomerular disease[MeSH Terms]) OR proteinuria[MeSH Terms]) OR renal insufficiency[MeSH Terms]) OR kidney disease[MeSH Terms]) OR chronic renal failure[MeSH Terms]) OR chronic kidney disease[MeSH Terms])	522,828
	#2	drug therapy, combination [MeSH Terms]	320,004
	#3	((renin–angiotensin system [MeSH Terms]) OR (angiotensin-converting enzyme inhibitor [MeSH Terms])) OR (angiotensin-receptor blocker [MeSH Terms])	45,299
	#4	#2 or #3	361,550
	#5	#1 and #4	18,664
Cochrane Library	#1	MeSH descriptor: [Diabetic Nephropathies] explode all trees	1,390
	#2	(hypertensive nephropathy): ti,ab,kw	344
	#3	(glomerular disease): ti,ab,kw	4,480
	#4	MeSH descriptor: [Proteinuria] explode all trees	2,231
	#5	MeSH descriptor: [Renal Insufficiency] explode all trees	8,664
	#6	MeSH descriptor: [Kidney Diseases] explode all trees	15,613
	#7	MeSH descriptor: [Kidney Failure, Chronic] explode all trees	4,573
	#8	MeSH descriptor: [Renal Insufficiency, Chronic] explode all trees	6,453
	#9	#1 or #2 or #3 or #4 or #5 or #6 or #7 or #8	19,593
	#10	MeSH descriptor: [Drug Therapy, Combination] explode all trees	43,531
	#11	MeSH descriptor: [Renin-Angiotensin System] explode all trees	796
	#12	MeSH descriptor: [Angiotensin-Converting Enzyme Inhibitors] explode all trees	3,965
	#13	(angiotensin-receptor blocker): ti,ab,kw	1,344

	#14	#10 or #11 or #12 or #13	48,181
	#15	#9 and #14	2,182
EMBASE	#1	'diabetic nephropathy'/exp OR 'hypertensive nephropathy'/exp OR 'glomerulopathy'/exp OR 'proteinuria'/exp OR 'kidney failure'/exp OR 'kidney disease'/exp OR 'chronic kidney failure'/exp	1,055,958
	#2	'combination drug therapy'/exp OR 'renin angiotensin aldosterone system'/exp OR 'angiotensin-converting enzyme inhibitor'/exp OR 'angiotensin receptor antagonist'/exp	405,894
	#3	#1 AND #2	66,187
	#4	#3 AND [randomized controlled trial]/lim	4,034
